# One-pot cross-enyne metathesis (CEYM)–Diels–Alder reaction of *gem*-difluoropropargylic alkynes

**DOI:** 10.3762/bjoc.9.305

**Published:** 2013-11-28

**Authors:** Santos Fustero, Paula Bello, Javier Miró, María Sánchez-Roselló, Günter Haufe, Carlos del Pozo

**Affiliations:** 1 Departamento de Química Orgánica, Universidad de Valencia, E-46100 Burjassot, Spain; 2 Laboratorio de Moléculas Orgánicas, Centro de Investigación Príncipe Felipe, E-46012 Valencia, Spain; 3 Organisch-Chemisches Institut, Westfälische Wilhelms-Universität, Corrensstraße 40, D-48149 Münster, Germany

**Keywords:** cross metathesis, Diels–Alder, one-pot reaction, organo-fluorine, propargylic difluorides

## Abstract

Propargylic difluorides **1** were used as starting substrates in a combination of cross-enyne metathesis and Diels–Alder reactions. Thus, the reaction of **1** with ethylene in the presence of 2^nd^ generation Hoveyda–Grubbs catalyst generates a diene moiety which in situ reacts with a wide variety of dienophiles giving rise to a small family of new fluorinated carbo- and heterocyclic derivatives in moderate to good yields. This is a complementary protocol to the one previously described by our research group, which involved the use of 1,7-octadiene as an internal source of ethylene.

## Introduction

In recent years the number of applications of olefin metathesis as a mild and competitive synthetic method for the creation of carbon–carbon bonds has exponentially increased, due to the availability of well-defined catalysts [1–3]. Particularly, enyne metathesis (EYM) is a powerful synthetic tool for generating 1,3-dienes by redistributing unsaturated functionalities between an alkene and an alkyne moiety via vinylalkylidene intermediates [4–6]. This is an atom economical process since it is an addition reaction and non-olefin byproducts are formed. Furthermore, a sequential use of EYM and Diels–Alder reactions generates highly functionalized carbo- and heterocyclic frameworks [7–11].

The intramolecular version of this process, the ring closing enyne metathesis (RCEYM) reaction, has found wide application, and several examples can be found in the literature [12–13]. However, the intermolecular version, i.e. the cross-enyne metathesis (CEYM) reaction, has been much less exploited probably due to its inherent problems of selectivity, which results in the formation of a mixture of *E*- and *Z*-isomers [14]. The discovery of the beneficial effect of ethylene has changed this tendency allowing the straightforward preparation of 1,3-dienes [15–16]. Thus, during the ethylene gas promoted CEYM reaction, ethylene does not incorporate into the product but intercepts the secondary metathesis pathways avoiding the formation of secondary products during the process [17–18].

Among organic fluorine compounds, propargylic fluorides constitute a relevant class of fluorinated building blocks. The transformational diversity of the alkynyl group converts them into versatile synthetic intermediates. Additionally, propargylic fluorides are prevalent motifs in life sciences, such as medicinal chemistry or crop protection. In this context, the preparation of monofluorinated propargylic compounds has been the subject of intense research, and efficient methodologies to access these derivatives have been devised [19–20]. However, the analogues bearing the *gem*-difluoro moiety next to unsaturated bonds have not received comparable attention, probably due to the limited availability of the starting materials. In this context, the recent introduction of difluoropropargyl bromide as fluorinated building block gave access to a wide variety of *gem*-difluoro-containing alkyne derivatives [21–22]. Recently, we have employed these fluorinated triple bond scaffolds in several types of cyclization reactions for the preparation of different difluoropropargylamides and ketones, having been subjected to intramolecular hydroaminations [23], cascade RCEYM–Diels–Alder reactions [24], [2 + 2 + 2] cycloadditions and gold-mediated dimerization reactions [25].

**Scheme 1:**
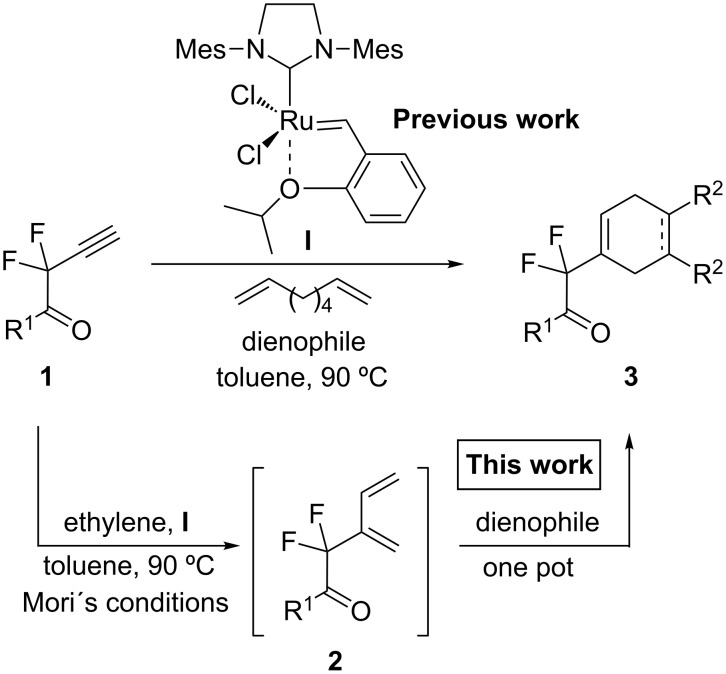
Sequential CEYM–Diels–Alder reaction.

Although the CEYM reaction has been found to be very fruitful for the preparation of 1,3-dienes, this protocol has remained almost unexplored for propargyl fluorides [26]. In this context, we have recently established a tandem multicomponent protocol CEYM–Diels–Alder reaction of several difluoropropargylic derivatives [27–28] mediated by 1,7-octadiene as an internal source of ethylene [29]. Following our ongoing interest in the use of these fluorinated building blocks, we decided to evaluate the CEYM reaction of several difluoropropargylamides and ketones in combination with a Diels–Alder reaction under Mori´s conditions, in order to compare this protocol with the aforementioned one (Scheme 1).

## Results and Discussion

**Scheme 2:**
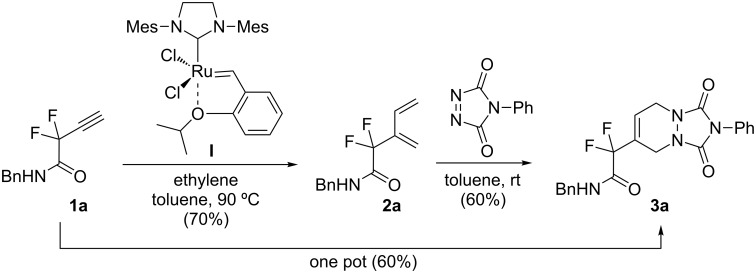
One-pot CM–Diels–Alder reaction with fluorinated alkyne **1a**.

In order to prove the efficiency of ethylene-mediated cross-enyne metathesis on our fluorinated alkynes, substrate **1a** was chosen as a model substrate. As expected, when a toluene solution of alkyne **1a** and 5 mol % of 2^nd^ generation Hoveyda–Grubbs catalyst **I** was heated under ethylene atmosphere (1 atm) for 2 h, the clean formation of diene **2a** was observed. This newly formed diene was isolated in 70% yield and it reacted smoothly with 4-phenyl-3*H*-1,2,4-triazole-3,5(4*H*)-dione as dienophile at room temperature to afford, after chromatographic purification, the corresponding Diels–Alder adduct **3a** in 60% yield (Scheme 2). It was interesting to find that this sequence can be performed as a one-pot procedure. Thus, when the formation of the diene intermediate **2a** was completed (determined by TLC), the dienophile was added to the reaction mixture and was allowed to react for two additional hours. Flash chromatography of the crude product afforded the desired tandem derivative **3a** in 60% overall yield (Scheme 2).

Next, the one-pot protocol was extended to other starting difluoropropargylic alkynes and dienophiles, affording a new family of carbo- and heterocyclic derivatives in moderate to good yields (Table 1).

**Table 1:** Preparation of compounds **3** by one-pot CEYM–Diels–Alder reaction of substrates **1** (method A).

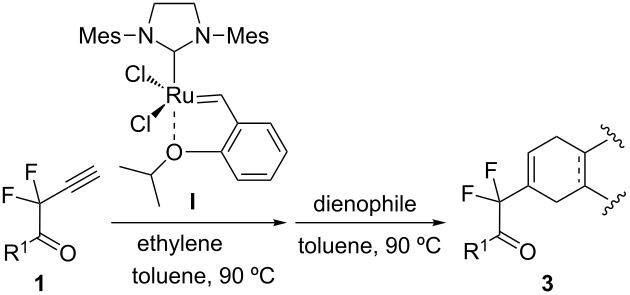						

entry	**1**	R^1^	dienophile	product	% yield method A^a^ (time, h)	% yield method B^b^ (time, h)

1	**1a**	Bn–NH	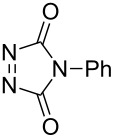	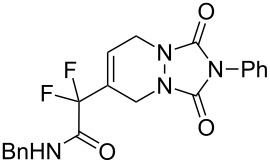 **3a**	60 (2 h)^c^	–
2	**1a**	Bn–NH	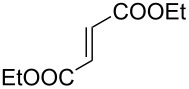	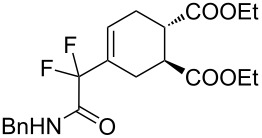 **3b**	55 (8 h)	70 (20 h)
3	**1a**	Bn–NH	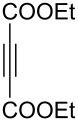	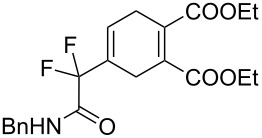 **3c**	51 (6 h)	55 (6 h)
4	**1a**	Bn–NH	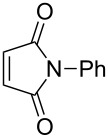	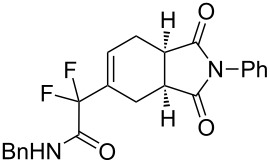 **3d**	50 (4 h)	63 (24 h)
5	**1a**	Bn–NH	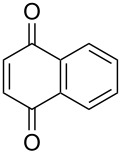	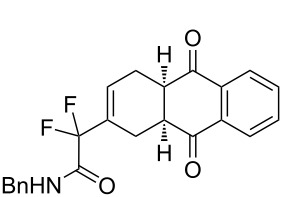 **3e**	47 (24 h)	50 (24 h)
6	**1a**	Bn–NH	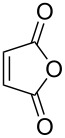	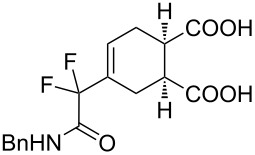 **3f**	85 (120 h)^c^	–
7	**1b**	(*R*)-Ph(Me)–CHNH	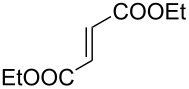	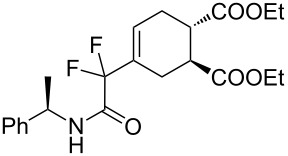 **3g** + 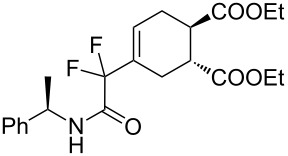 **3g’**	87 (4 h)^d^	73 (24 h)^d^
8	**1b**	(*R*)-Ph(Me)–CHNH	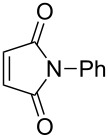	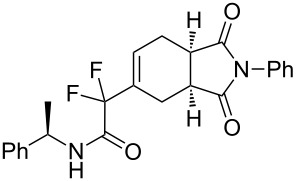 **3h** + 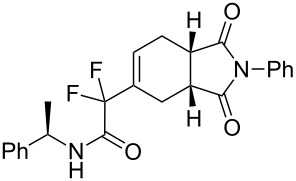 **3h’**	74 (2 h)^d^	70 (10 h)^d^
9	**1b**	(*R*)-Ph(Me)–CHNH	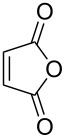	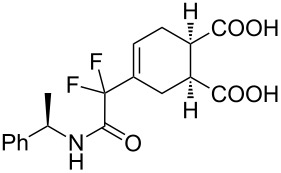 **3i** + 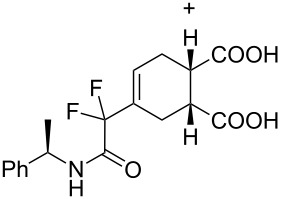 **3i’**	74 (20 h)^c,d^	–
10	**1b**	(*R*)-Ph(Me)–CHNH	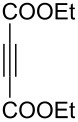	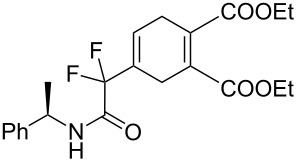 **3j**	63 (10 h)	60 (24 h)
11	**1c**	PropylNH	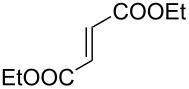	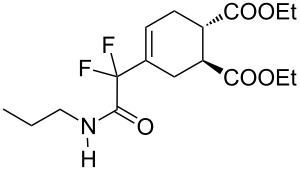 **3k**	40 (5 h)	–
12	**1d**	Ph	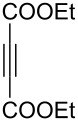	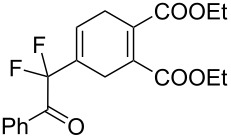 **3l**	48 (4 h)	55 (15 h)
13	**1d**	Ph	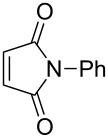	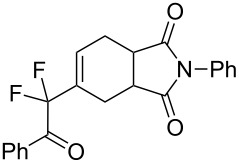 **3m**	28 (8 h)	–
14	**1d**	Ph	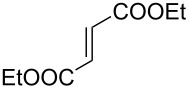	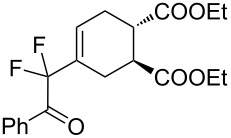 **3n**	58 (12 h)	70 (17 h)
15	**1e**	Cy	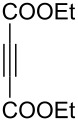	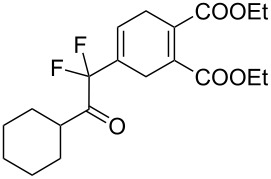 **3o**	54 (12 h)	–

^a^Method A: One-pot protocol with Mori´s conditions. The formation of the corresponding diene **2** with ethylene was complete after 2 h at 90 °C for all substrates. ^b^Method B: Tandem multicomponent protocol mediated by 1,7-octadiene [29]. ^c^When maleic anhydride or 4-phenyl-3*H*-1,2,4-triazole-3,5(4*H*)-dione were used as dienophiles, the Diels–Alder reaction was performed at rt. With maleic anhydride final products were isolated as the corresponding diacid derivatives. ^d^In all cases, adducts were obtained as an inseparable 1:1 mixture of diastereoisomers.

A wide variety of nucleophiles is compatible with the one-pot protocol (Table 1, method A, entries 1–6). Ethyl fumarate gave the desired product **3b** in 55% yield (Table 1, method A, entry 2). It is interesting to point out that diethyl acetylenedicarboxylate (DEAD) afforded adduct **3c** in 51% yield, and no aromatization was observed during the process (Table 1, method A, entry 3). When maleic anhydride was used as dienophile, the corresponding diacid **3f**, arising from the anhydride ring opening under the reaction conditions, was observed as the major product (Table 1, method A, entries 6 and 9). With chiral starting material **1b**, in all cases a 1:1 mixture of diastereoisomers was obtained which could not be separated. This indicates that the chiral information is not close enough to the reacting centre (Table 1, method A, entries 7–10). Finally, fluorinated ketones could also be used as substrates for the sequential process (Table 1, method A, entries 12–15) and again with alkynes as dienophiles, no aromatization of the final products was detected (Table 1, method A, entry 12).

The yields that appear in Table 1 in the last column (method B) represent the reaction performed under the tandem-multicomponent conditions mediated by 1,7-octadiene (Scheme 1). In general, yields are comparable using either methodology, indicating that they are applicable for the synthesis of new carbo- and heterocyclic derivatives bearing a *gem*-difluoro moiety in an efficient manner. However, at this point it is important to mention that when the 1,7-octadiene protocol was applied using maleic anhydride or 4-phenyl-3*H*-1,2,4-triazole-3,5(4*H*)-dione as dienophiles (Table 1, method B, entries 1, 6 and 9), a complex mixture was obtained. This is probably due to the fact that under those thermal conditions, it is not possible to use these types of dienophiles since they decompose while being heated. The sequential generation of the dienic intermediate **2** and the Diels–Alder reaction allow performing the second step at rt, avoiding these problems. Thus, although a tandem-multicomponent protocol is more desirable, the use of the one-pot protocol expand the utility and scope of this methodology, since milder conditions can be employed in the cyclization step.

## Conclusion

In conclusion, a tandem one-pot enyne-cross metathesis-Diels–Alder reaction of difluoropropargylic alkynes with a variety of dienophiles has been described. The process took place in moderate to good yields, giving rise to a new family of fluorinated carbo- and heterocyclic derivatives in a very simple manner. In comparison with the tandem multicomponent protocol, the one-pot sequence is a complementary methodology, since although comparable yields of the final adducts can be obtained, this methodology is compatible with a greater variety of dienophiles.

## Experimental

**General experimental methods.** Reactions were carried out under argon atmosphere unless otherwise indicated. The solvents were purified prior to use: THF, diethyl ether and toluene were distilled from sodium/benzophenone; dichloromethane and acetonitrile were distilled from calcium hydride. The reactions were monitored with the aid of thin-layer chromatography (TLC) on 0.25 mm precoated silica gel plates. Visualization was carried out with UV light and aqueous ceric ammonium molybdate solution or potassium permanganate stain. Flash column chromatography was performed with the indicated solvents on silica gel 60 (particle size 0.040–0.063 mm). ^1^H and ^13^C NMR spectra were recorded on 300 or 400 MHz spectrometers. Chemical shifts are given in ppm (δ), with reference to the residual proton resonances of the solvents. Coupling constants (*J*) are given in Hertz (Hz). The letters m, s, d, t, and q stand for multiplet, singlet, doublet, triplet and quartet, respectively. The letters br indicate that the signal is broad. Starting fluorinated amides **1** [23–25] and compounds **3b,c,e,g,h,j,l,n** [29] were previously described.

**General procedure for the one-pot process.** Ethylene was bubbled through a solution of catalyst **I** (5 mol %) in dry toluene (2.4 mL) for 3 minutes at room temperature in a sealed tube. Substrate **1** (0.12–0.25 mmol) was added next and it was heated at 90 °C for 2 hours. Once the intermediate diene was formed (by TLC), it was cooled to room temperature and the corresponding dienophile was added. The reaction mixture was stirred at temperatures and times given in Table 1. Finally, after removal of solvents, the reaction mixture was purified by flash chromatography in hexanes/ethyl acetate (3:1).

***N*-Benzyl-2,2-difluoro-3-methylenepent-4-enamide (2a).** Following the procedure described above and before adding the dienophile, the crude mixture was subjected to flash chromatography affording 41 mg of **2a** (70% yield) as a yellow oil starting from 52 mg of **1a**. ^1^H NMR (CDCl_3_, 300 MHz) δ 4.52 (d, *J* = 5.8 Hz, 2H), 5.25 (d, *J* = 11.3 Hz, 1H), 5.52 (d, *J* = 17.9 Hz, 1H), 5.64 (d, *J* = 15.9 Hz, 2H), 6.32 (dd, *J*_1_ = 11.3 Hz, *J*_2_ = 17.7 Hz, 1H), 6.65 (br s, 1H), 7.26–7.39 (m, 5H); ^13^C NMR (CDCl_3_, 300 MHz) δ 43.61, 114.40 (t, ^1^*J*_CF_ = 253.2 Hz), 118.17, 119.33 (t, ^3^*J*_CF_ = 8.6 Hz), 127.81, 127.93, 128.84, 131.02 (t, ^3^*J*_CF_ = 2.3 Hz), 136.75, 138.82 (t, ^2^*J*_CF_ = 22.3 Hz), 163.23 (t, ^2^*J*_CF_ = 29.9 Hz); ^19^F NMR (CDCl_3_, 282 MHz) δ −105.57 (s, 2F); HRMS: [M + 1]^+^ calcd for C_13_H_14_F_2_NO, 238.1038; found, 238.1042.

***N*-Benzyl-2-(1,3-dioxo-2-phenyl-2,3,5,8-tetrahydro-1*H*-[1,2,4]triazolo[1,2*a*]pyridazin-6-yl)-2,2-difluoroacetamide (3a).** Following the general procedure described above, 61 mg of **3a** (60% yield) were obtained as a white solid starting from 52 mg of **1a**. mp = 148–150 °C; ^1^H NMR (CDCl_3_, 300 MHz) δ 4.26–4.29 (m, 2H), 4.34 (s, 2H), 4.5 (d, *J* = 5.7 Hz, 2H), 6.46 (m, 1H), 6.86 (br s, 1H), 7.16–7.53 (m, 10H); ^13^C NMR (CDCl_3_, 300 MHz) δ 41.8 (t, ^3^*J*_CF_ = 4 Hz), 43.1, 43.8, 113.3 (t, ^1^*J*_CF_ = 253.8 Hz), 123.7 (t, ^3^*J*_CF_ = 8.7 Hz), 125.4, 126.8 (t, ^2^*J*_CF_ = 25.2 Hz), 127.9, 128.2, 128.4, 129.0, 129.2, 130.8, 136.3, 152.3, 152.4, 162.2 (t, ^2^*J*_CF_ = 29.9 Hz); ^19^F NMR (CDCl_3_, 282 MHz) δ −106.9 (s, 2F); HRMS: [M]^+^ calcd for C_21_H_18_F_2_N_4_O_3_, 412.1347; found, 412.1344.

**Diethyl 4-(2-(benzylamino)-1,1-difluoro-2-oxoethyl)cyclohex-4-ene-1,2-dicarboxylate (3b).** Following the general procedure described above, 30 mg (55% yield) of **3b** were obtained as a white solid starting from 26 mg of **1a**.

**Diethyl 4-(2-(benzylamino)-1,1-difluoro-2-oxoethyl)cyclohexa-1,4-diene-1,2-dicarboxylate (3c).** Following the general procedure described above, 52 mg (51% yield) of **3c** were obtained as a dark brown oil starting from 52 mg of **1a**.

***N*-Benzyl-2-(1,3-dioxo-2-phenyl-2,3,3a,4,7,7a-hexahydro-1*H*-isoindol-5-yl)-2,2-difluoroacetamide (3d).** Following the general procedure described above, 25 mg of **3d** (50% yield) were obtained as a dark brown oil starting from 26 mg of **1a**. ^1^H NMR (CDCl_3_, 300 MHz) δ 2.32–2.47 (m, 2H), 2.82–2.93 (m, 2H), 3.27–3.38 (m, 2H), 4.46 (d, *J* = 5.7 Hz, 2H), 6.49–6.55 (m, 1H), 6.68 (br s, 1H), 7.27–7.47 (m, 10H); ^13^C NMR (CDCl_3_, 300 MHz) δ 22.8, 23.9, 38.7, 39.1, 43.7, 114.2 (t, ^1^*J*_CF_ = 251.0 Hz), 126.5, 127.9, 128.0, 128.7, 128.9, 129.1, 129.9 (t, ^3^*J*_CF_ = 8.9 Hz), 131.8, 131.9 (t, ^2^*J*_CF_ = 24.4 Hz), 136.6, 162.8 (t, ^2^*J*_CF_ = 30.1 Hz), 177.7, 178.1; ^19^F NMR (CDCl_3_, 282 MHz) δ −106.5 (d, *J*_FF_ = 258.1 Hz, 1F), −108.7 (d, *J*_FF_ = 258.4 Hz, 1F); HRMS: [M]^+^ calcd for C_23_H_20_F_2_N_2_O_3_, 410.1442; found, 410.1445.

***N*-Benzyl-2-(9,10-dioxo-1,4,4a,9,9a,10-hexahydroanthracen-2-yl)-2,2-difluoroacetamide (3e).** Following the general procedure described above, 24 mg of **3e** (47% yield) were obtained as a dark brown oil starting from 26 mg of **1a**.

**4-[2-(Benzylamino)-1,1-difluoro-2-oxoethyl]cyclohex-4-ene-1,2-dicarboxylic acid (3f).** Following the general procedure described above, 71 mg of **3f** (85% yield) were obtained as a dark brown oil starting from 52 mg of **1a**. ^1^H NMR (CDCl_3_, 300 MHz) δ 2.46–2.52 (m, 2H), 2.68–2.75 (m, 2H), 3.07 (m, 1H), 3.17 (m, 1H), 4.47 (d, *J* = 5.7 Hz, 2H), 6.21 (s, 1H), 6.80–6.84 (m, *J* = 5.4 Hz, 1H), 7.25–7.37 (m, 5H), 7.77 (br s, 2H); ^13^C NMR (CDCl_3_, 300 MHz) δ 23.1, 25.0, 38.5, 38.9, 43.6, 114.8 (t, ^1^*J*_CF_ = 251.1 Hz), 127.8, 127.9, 128.8, 136.6, 163.6 (t, ^2^*J*_CF_ = 30.6 Hz), 178.3, 178.5; ^19^F NMR (CDCl_3_, 282 MHz) δ −117.0 (d, *J*_FF_ = 258.9 Hz, 1F), −118.0 (d, *J*_FF_ = 258.7 Hz, 1F); HRMS: [M + Na]^+^ calcd for C_17_H_17_F_2_NO_5_, 376.0972; found, 376.0969.

**Diethyl 4-(1,1-difluoro-2-oxo-2-[(*R*)-1-phenylethylamino)ethyl]cyclohex-4-ene-1,2-dicarboxylate (3g + 3g’).** Following the general procedure described above, 41 mg (87% yield) of a inseparable mixture of **3g** and **3g’** were obtained as a dark brown oil starting from 25 mg of **1b**.

**2-(1,3-Dioxo-2-phenyl-2,3,3a,4,7,7a-hexahydro-1*H*-isoindol-5-yl)-2,2-difluoro-*N*-[(*R*)-1-phenylethyl]acetamide (3h + 3h’).** Following the general procedure described above, 34 mg (74% yield) of a inseparable mixture of **3h** and **3h’** were obtained as a dark brown solid starting from 25 mg of **1b**.

**4-[1,1-difluoro-2-oxo-2-((*R*)-1-phenylethylamino)ethyl]cyclohexa-4-ene-1,2-dicarboxylate (3i + 3i’).** Following the general procedure described above, 24.3 mg of an inseparable mixture of **3i** and **3i’** were obtained as a dark brown oil starting from 25 mg of **1b. **^1^H NMR (CDCl_3_, 300 MHz) δ1.53 (dd, *J* = 6.9 Hz, *J* = 4.8 Hz, 3H), 2.46–2.51 (m, 2H), 2.67–2.74 (m, 2H), 3.02–3.07 (m, 1H), 3.15–3.17 (m, 1H), 5.08–5.17 (m, 1H), 6.17 (d, *J* = 13.5 Hz, 1H), 6.63–6.68 (br m, 1H), 7.28–7.38 (m, 5H); ^13^C NMR (CDCl_3_, 300 MHz) δ 21.2, 23.4, 25.2, 49.2, 114.7 (t, ^1^*J*_CF_ = 255.0 Hz), 126.1, 127.7, 128.7, 141.7, 162.5 (t, ^2^*J*_CF_ = 30.7 Hz), 177.6, 177.9; ^19^F NMR (CDCl_3_, 282 MHz) δ −106.6 (d, *J*_FF_ = 258.9 Hz, 1F), −106.8 (d, *J*_FF_ = 256.4Hz, 1F), −107.8 (d, *J*_FF_ = 255.6 Hz, 1F), −107.9 (d, *J*_FF_ = 257.5 Hz, 1F); HRMS: [M + Na]^+^ calcd for C_18_H_19_F_2_NO_5_, 390.1129; found, 390.1133.

**(*R*)-Diethyl 4-[1,1-difluoro-2-oxo-2-(1-phenylethylamino)ethyl]cyclohexa-1,4-diene-1,2-dicarboxylate (3j).** Following the general procedure described above, 59.5 mg (63% yield) of **3j** were obtained as a dark brown oil starting from 50 mg of **1b** [29].

**Diethyl 4-[1,1-difluoro-2-oxo-2-(propylamino)ethyl]cyclohex-4-ene-1,2-dicarboxylate (3k).** Following the general procedure described above, 27 mg of **3k** (40% yield) were obtained as a dark brown oil starting from 30 mg of **1c**. ^1^H NMR (CDCl_3_, 300 MHz) δ 0.93 (t, *J* = 7.5 Hz, 3H), 1.23 (t, *J* = 6.9 Hz, 3H), 1.24 (t, *J* = 7.2 Hz, 3H), 1.57 (m, 2H), 2.22–2.37 (m, 2H), 2.50–2.62 (m, 2H), 2.86 (m, 2H), 3.27 (q, *J* = 6.0 Hz, 2H), 4.13 (q, *J* = 6.9 Hz, 4H), 6.18–6.20 (br m, 1H); ^13^C NMR (300 MHz) δ 11.2, 14.0, 22.4, 25.0 (t, ^4^*J*_CF_ = 2.3 Hz), 27.3, 40.4, 40.6, 41.2, 60.8, 60.9, 114.7 (t, ^1^*J*_CF_ = 250.9 Hz), 127.6 (t, ^3^*J*_CF_ = 8.6 Hz), 129.0 (t, ^2^*J*_CF_ = 24.1 Hz), 163.2 (t, ^2^*J*_CF_ = 30.2 Hz), 173.7, 173,9; ^19^F NMR (CDCl_3_, 282 MHz) δ −106.8 (d, *J*_FF_ = 257.8 Hz, 1F), −107.9 (d, *J*_FF_ = 258.4 Hz, 1F); HRMS: [M + 1]^+^ calcd for C_17_H_26_F_2_NO_5_, 362.1774; found, 362.1779.

**Diethyl 4-(1,1-difluoro-2-oxo-2-phenylethyl)cyclohexa-1,4-diene-1,2-dicarboxylate (3l).** Following the general procedures described above, 20 mg (48% yield) of **3l** were obtained as a dark brown oil starting from 20 mg of **1d**.

**5-(1,1-Difluoro-2-oxo-2-phenylethyl)-2-phenyl-3a,4,7,7a-tetrahydro-1*H*-isoindole-1,3(2*H*)-dione (3m).** Following the general procedure described above, 15 mg of **3m** (28% yield) were obtained as a dark brown oil starting from 25 mg of **1d**. ^1^H NMR (CDCl_3_, 300 MHz) δ 2.26–2.41 (m, 2H), 2.73–2.84 (m, 2H), 3.18–3.30 (m, 2H), 6.33–6.38 (m, 1H), 7.09–7.14 (m, 3H), 7.23–7.35 (m, 5H), 7.47–7.52 (m, 1H), 7.91–7.94 (m, 2H); ^13^C NMR (CDCl_3_, 300 MHz) δ 23.2 (t, ^3^*J*_CF_ = 2.6 Hz), 23.8, 38.8, 39.0, 116.2 (t, ^1^*J*_CF_ = 253.0 Hz), 126.4, 128.6, 128.7, 129.1, 129.7 (t, ^3^*J*_CF_ = 8.9 Hz), 130.2 (t, ^4^*J*_CF_ = 3.0 Hz), 131.8, 133.2 (t, ^2^*J*_CF_ = 23.9 Hz), 134.4, 177.7, 178.2, 188.3 (t, ^2^*J*_CF_ = 32.1 Hz); ^19^F NMR (CDCl_3_, 282 MHz) δ −112.367 (d, *J*_FF_ = 284.6 Hz, 1F), −111.2 (d, *J*_FF_ = 284.4 Hz, 1F); HRMS: [M + 1]^+^ calcd for C_22_H_18_F_2_NO_3_, 382.1255; found, 382.1258.

**Diethyl 4-[1,1-difluoro-2-oxo-2-(phenylamino)ethyl]cyclohex-4-ene-1,2-dicarboxylate (3n).** Following the general procedure described above, 24.5 mg (58% yield) of **3n** were obtained as a yellow oil starting from 20 mg of **1d**.

**Diethyl 4-(2-cyclohexyl-1,1-difluoro-2-oxoethyl)cyclohexa-1,4-diene-1,2-dicarboxylate (3o).** Following the general procedure described above, 28 mg of **3o** (54% yield) were obtained as a dark oil starting from 25 mg of **1e**. ^1^H NMR (CDCl_3_, 300 MHz) δ 1.11–1.41 (m, 5H), 1.63–1.84 (m, 5H), 2.30–2.46 (m, 5H), 2.71–2.92 (m, 3H), 3.26–3.36 (m, 2H), 6.37–6.41 (m, 1H), 7.25–7.29 (m, 2H), 7.35–7.49 (m, 3H); ^13^C NMR (300 MHz) δ 30.0 (t, ^4^*J*_CF_ = 2.7 Hz), 23.8, 25.3, 25.4, 28.3, 38.7, 45.1, 115.1 (t, ^1^*J*_CF_ = 253.7 Hz), 126.4, 128.6, 129.1, 129.5 (t, ^3^*J*_CF_ = 9.1 Hz), 132.2 (t, ^2^*J*_CF_ = 24.2 Hz), 177.7, 178.2, 202.7 (t, ^2^*J*_CF_ = 31.7 Hz); ^19^F NMR (CDCl_3_, 282 MHz) δ −109.9 (d, *J*_FF_ = 276.7 Hz, 1F), −106.0 (d, *J*_FF_ = 276.7 Hz, 1F); HRMS: [M]^+^ calcd for C_22_H_23_F_2_NO_3_, 387.1646; found, 387.1656.
